# Heparin treatment mitigates radiation-induced oral mucositis in mice by interplaying with repopulation processes

**DOI:** 10.1007/s00066-018-01423-4

**Published:** 2019-01-28

**Authors:** M. Kowaliuk, I. Schröder, P. Kuess, W. Dörr

**Affiliations:** 1 Department of Radiation Oncology—ATRAB-Applied and Translational Radiobiology, Department of Radiation Oncology, Medical University of Vienna, Währinger Gürtel 18–20, 1090 Vienna, Austria; 2 Christian Doppler Laboratory for Medical Radiation Research for Radiation Oncology, Medical University of Vienna, Vienna, Austria; 3 IMC FH Krems, University of Applied Sciences, Krems, Austria

**Keywords:** Normal tissue effects, Radiotherapy, Animal model, Oral epithelium, Normalgewebsreaktionen, Strahlentherapie, Tiermodel, Mundschleimhaut

## Abstract

**Purpose:**

To investigate the mechanistic background of the muco-protective effect of systemic heparin treatment on the development of radiation-induced oral mucositis in mice.

**Materials and methods:**

Fractionated irradiation was given to the snouts of male C3H/Neu mice over 2 weeks (10 × 3 Gy), either alone or in combination with daily subcutaneous application of unfractionated or low molecular weight heparin (40 or 200 I.U./mouse, respectively). Over this course of 14 days, groups of mice (*n* = 3) were sacrificed every second day, their tongues excised and processed for histological analysis. The epithelial radiation response with and without heparin treatment was evaluated in terms of tissue morphology, proliferation and expression of cell contact molecules.

**Results:**

Systemic treatment with heparins significantly reduced the cellular effects of irradiation to the oral epithelium. Heparin treated animals showed significantly higher total epithelial cell numbers and thickness throughout the study course. Bromodeoxyuridine (BrdU) incorporation analyses revealed that markedly more epithelial cells retained their proliferative capacity in the beginning of the first treatment week, but the proliferation of the mucosa was not stimulated during the rest of the study course. The expression of the adherens junction protein β‑catenin was slightly elevated in heparin treated animals, on day 2 the increase was statistically significant. The expression of e‑cadherin and occludin was mostly unaffected by the concomitant heparin treatment.

**Conclusion:**

The findings of this study indicate an interplay of additional heparin treatment with the repopulation processes, leading to an earlier onset of this adaptive radiation response in oral mucosa. Importantly, we could demonstrate that the protective potential of heparin did not rely on stimulation of normal tissue proliferation. Since both heparin preparations are already approved for clinical use, they are considered as promising candidates for future clinical studies.

## Abstract (de)

**Ziel:**

Untersucht werden sollten die mechanistischen Hintergründe des protektiven Effekts einer systemischen Heparingabe auf die Ausprägung der radiogenen oralen Mukositis in der Maus.

**Material und Methoden:**

Fraktionierte Bestrahlung wurde auf die Schnauzen von männlichen C3H/Neu-Mäusen über 2 Wochen (10 × 3 Gy) appliziert, entweder allein oder in Kombination mit täglicher s.c. Gabe von unfraktioniertem bzw. niedermolekularem Heparin (40 bzw. 200 I.E./Maus). Über diese Zeit wurden jeden zweiten Tag Gruppen von Mäusen (*n* = 3) euthanasiert, ihre Zungen wurden entnommen und für die histologischen Untersuchungen aufbereitet. Die epitheliale Strahlenreaktion mit bzw. ohne Heparinbehandlung wurde hinsichtlich der Gewebemorphologie, der Proliferation und der Expression von Zellkontaktmolekülen untersucht.

**Ergebnisse:**

Eine systemische Heparingabe reduzierte signifikant den zellulären Effekt der Bestrahlung auf das orale Epithel. Über den ganzen Behandlungszeitraum hinweg wiesen mit Heparin behandelte Mäuse signifikant höhere Gesamtzellzahlen und Schichtdicken des Epithels auf. Bromdesoxyuridin(BrdU)-Inkorporationsstudien ergaben, dass deutlich mehr Epithelzellen ihre proliferative Kapazität während der ersten Behandlungswoche beibehalten hatten. Während der restlichen Behandlungszeit wurde keine zusätzliche Stimulation der Proliferation im Epithel beobachtet. Die Expression des Zellkontaktmoleküls β‑Catenin war infolge der Heparinbehandlung leicht erhöht, an Tag 2 konnte ein signifikanter Unterschied beobachtet werden. Die Expression von e‑Cadherin und Occludin wurde durch die begleitende Heparinbehandlung nicht beeinflusst.

**Schlussfolgerung:**

Die Ergebnisse dieser Studie deuten auf eine Interaktion der Heparingabe mit den Repopulierungsprozessen hin, was ein früheres Einsetzen dieser adaptiven Strahlenreaktion in der oralen Mukosa zur Folge hat. Zudem konnte gezeigt werden, dass der protektive Effekt der Heparingabe nicht auf der Stimulation der Proliferation im Normalgewebe basierte. Da beide Heparine bereits in der Klinik zugelassen sind, stellen sie geeignete Kandidaten für weiterführende klinische Studien dar.

## Background

Oral mucositis is the most frequently occurring early side effect of radiotherapy in patients treated for head-and-neck cancers. The majority of patients undergoing curative treatment develop a severe reaction, manifesting as painful confluent lesions and/or ulcerations of the epithelial membranes lining the oral cavity [1]. Oral mucositis significantly reduces the patient’s quality of life due to mucositis-associated pain and speaking and swallowing difficulties, which often necessitate parenteral nutrition. Additionally, radiation-induced oral mucositis often leads to local and systemic infections because of disturbed mucosal barrier function. Besides the socio-economic factor due to mucositis-associated hospitalizations [2], these radiation-induced complications might lead to treatment interruptions thus significantly lowering the tumor control probability.

However, current treatment strategies are purely symptomatic, based on the improvement of oral hygiene and pain relief [3]. So far, the only biology-based treatment approach against oral mucositis is palifermin and the recommended administration is limited to myoablative treatment only. Being a recombinant human keratinocyte growth factor (KGF), the question regarding the proliferation stimulating effect on the malignant cells is still of concern and, in combination with its high costs, prevents the use of palifermin in head-and-neck patients [4].

Oral mucosa represents a turn-over tissue, with a strictly regulated equilibrium between cell proliferation in the germinal and cell loss in the superficial layer [5]. Radiation negatively impacts epithelial proliferation. Since cell loss and shedding is not affected by radiation and continues at its physiological rate, radiotherapy results in epithelial hypoplasia and consequently in complete denudation. This represents the main cause of oral mucositis and causes the loss of the barrier function of the oral epithelium against potentially toxic substances and bacteria [6]. The integrity of the mucosal barrier is maintained via tight and adherence junction complexes [7]. Tight junctions are formed by the proteins claudin and occludin and, besides the tissue barrier function, play a role in transport of water and small molecules between the cells by forming size selective channels [8]. Adherence junctions are formed by the transmembrane proteins e‑cadherin and α/β-catenin and are responsible for the interaction of the cell with the actin of the cytoskeleton [9]. Thus, they act as cellular anchors and regulate the cell–cell and cell–extracellular matrix interaction, additionally maintaining the polarization of the cell. Besides the above mentioned mechanical properties of the cell contact molecules, they play a role in signaling within and between the cells [10]. Junction complexes thus are involved not only in physiological processes such as growth, proliferation and migration but also in pathological conditions such as wound healing and inflammation. A study published in 2018 by Gruber et al. showed that the expression of epithelial junctions is upregulated in the oral mucosa of mice in response to fractionated irradiation, thus representing a novel parameter of the epithelial radiation response [11].

Recently, we described a protective and mitigative effect of systemic heparin administration for radiation-induced mucositis in mice [12]. Heparin belongs to the group of glycosaminoglycans (GAGs) which represent a polydisperse, heterogeneous mixture of molecules with variable chain lengths, disaccharide composition and sulfation status [13]. In clinical practice, heparins have been used for over eighty years due to their anticoagulant activity. The function of the biologically available heparin, which is released by the mast cells during inflammatory events, is still not clear [14]. Nowadays, a great body of literature exists proposing various binding partners for exogenously supplied heparins, including growth factors, extracellular matrix (ECM) proteins, cytokines and chemokines. Thus, apart from coagulation, heparins might play a pivotal role in the processes of proliferation, development, inflammation, infection and wound healing [15].

Therefore, the present study was initiated to analyze the mechanistic background of the radio-protective effect of the systemic heparin treatment. Here, we particularly focus on heparin’s impact on changes in epithelial morphological parameters such as total epithelial cell number and thickness, and cell proliferation, represented by incorporation of bromodeoxyuridine (BrdU). To analyze the effect of heparin treatment on the integrity of oral mucosa, the expression of epithelial tight (occludin) and adherence (e-cadherin and β‑catenin) junction proteins will be evaluated.

## Materials and methods

### Animals and housing

Twelve to 14 weeks old male mice of the inbred C3H/Neu strain from the breeding facility of the Department for the Biomedical Research of the Medical University of Vienna were used in the described experiments. Mice were bred and housed under specified pathogen-free conditions with controlled temperature (20–22 °C), humidity (45–55%) and a 12/12-h light–dark rhythm. Free access to standard mouse diet (Sniff Spezialdiäten GmbH, Soest, Germany) and filtered water was provided. A maximum of five animals were kept in IVC cages (Tecniplast®, Buguggiate, Italy) on aspen wood bedding (ABEDD®, Lab & Vet Service GmbH, Köflach, Austria).

### Irradiation technique

Fractionated irradiation with 3 Gy per fraction was applied to the whole snouts of the animals. For this, un-anesthetized mice were guided into a perspex tube (28 mm inner diameter). A conical hole at the front end of the tube served for the positioning of the animal. The rear end was closed to prevent the escaping of the animals. Up to eight mice were irradiated simultaneously. The snout of the animals, including the entire tongue, was irradiated; the rest of the body shielded by a lead equivalent.

Irradiation was performed using an YXLON MG325 X‑ray device (YXLON International GmbH, Hamburg, Germany) with a vertical beam. The X‑ray unit was operated with a tube voltage of 200 kV and a tube current of 20 mA. The dose rate was regularly controlled and accounted at the focus-to-surface distance of 45.5 cm approximately 1 Gy/min. In addition to the inherent filtration with 3 mm Be, 4 mm Al and 0.6 mm Cu beam filters were used. The commissioning of the irradiation set-up revealed dose homogeneity between the individual snout positions of ±3%.

### Heparins

Fractionated irradiation was combined with daily application of heparins: unfractionated heparin (UFH, heparin medicamentum; medicamentum pharma, Allerheiligen im Mürztal, Austria) or low molecular-weight heparin Lovenox® (LMWH, enoxaparin sodium; Sanofi, Paris, France). Heparins were diluted in saline and injected subcutaneously at a daily dose of 40 IU/mouse for UFH and 200 IU/mouse for LMWH, respectively, with the injection volume not exceeding 150 µL. The drug was applied two hours after irradiation and on days without irradiation approximately at the same time of day.

### Experimental design

**Fig. 1:**
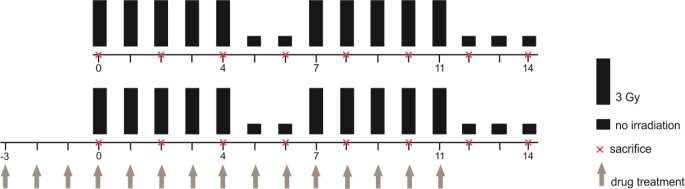
Experimental design. Daily fractionated irradiation was applied in fractions of 3 Gy over two weeks (10 × 3 Gy) including an irradiation-free weekend either alone or in combination with daily heparin treatment. UFH or LMWH (40 and 200 U/mouse/day, respectively) was applied subcutaneously starting three days before the first fraction (day −3) until the last fraction on day 11. Groups of 3 animals were sacrificed every second day from day 0 until day 14. *UFH* unfractionated heparin; *LMWH* low-molecular-weight heparin

Fractionated irradiation was given in fractions of 3 Gy on five consecutive days per week followed by an irradiation free weekend. The maximum number of applied fractions was 10 (days 0–4, 7–11). The control experiment comprised irradiation only. When fractionated irradiation was combined with drug administration, either UFH or LMWH was given daily starting three days before the first fraction (day −3) until the last irradiation day (day 11). Starting at day 0, groups of three animals were sacrificed every second day, their tongues excised and fixed in paraformaldehyde for further histological analysis. On days of sacrifice, no irradiation or drug were applied. Non-irradiated and untreated mice served as controls. The overview of the study design is given in Fig. 1, summarizing the irradiation and drug administration scheme.

### Immunohistochemical procedures

Excised tongues were fixed in 4% paraformaldehyde for max. 48 h, separated in halves along the median line and subjected to standard paraffin embedding. Sample sections of 4 µm were deparaffinized and rehydrated using xylene and a graded alcohol series. Heat mediated antigen retrieval in citrate buffer, pH 6, was performed by boiling the sections in a microwave at 640 W for 20 min. Endogenous peroxidase activity was blocked by incubation with 3% H_2_O_2_ for 10 min. Subsequent staining procedures were performed using a Vectastain® ABC kit (Vector Laboratories, Burlingame, CA, USA). After blocking of unspecific binding sites by 2% normal serum for 1 h at room temperature, primary antibodies were applied and incubated over night at 4 °C. The secondary antibody was added for 1 h at room temperature. Then, sections were treated with the avidin–biotin complex for 30 min at room temperature. The binding of the primary antibody was visualized by 3,3-diaminobenzidine (DAB) chromogen (Vector Laboratories, Burlingame, CA, USA) and hematoxylin nuclear counterstain. Slices were dehydrated using an alcohol series, cleared with xylene and cover-slipped.

Primary antibodies were purchased from Abcam® (Cambridge, MA, USA), diluted in TBS and used at following concentrations: anti-BrdU (Cat. No. 6326; rat polyclonal) 1:1000; anti-β-catenin (Cat. No. 32572; rabbit monoclonal) 1:300; anti-e-cadherin (Cat. No. 76319; rabbit monoclonal) 1:200; anti-occludin (Cat. No. 64482; rabbit polyclonal) 1:70.

### Histological analysis

Histological analysis was performed using an Olympus light microscope at 400× magnification with help of an optical grid. Epithelial cellularity and thickness as well as fractions of cells expressing targeted markers were evaluated in the epithelium of the lower mouse tongue in at least 5 microscopic fields (250 µm per field). The number of nucleated cells was assessed separately in the proliferative germinal and post-mitotic functional layers, based on the different morphology of these cellular compartments.

The total epithelial cell number was calculated as the sum of the germinal and functional values. The total cell number of the control specimen was set to 100% and the values of the irradiated and drug treated samples were calculated in relation to the control values. The epithelial thickness was evaluated using an optical grid.

BrdU incorporation and expression of cell junction proteins was assessed by calculating the fraction of marker-positive cells. Additionally, the respective staining intensity corresponding to the amount of the incorporated BrdU/expressed protein, was evaluated semi-quantitatively by assigning a score of arbitrary units (a.u.) from 0 (no), 1 (weak), 2 (intermediate) to 3 (strong signal) to each examined microscopic field.

### Statistical analysis

Statistical analysis was performed using the GraphPad Prism statistical software (GraphPad Software Inc., La Jolla, CA, USA). For each animal, the mean value and the standard deviation (SD) was calculated; based on these values, the mean and the standard deviation of each experimental group could be determined. To test for the significant difference between the mean values of the groups the two-way ANOVA was conducted. To correct for multiple comparisons using statistical hypothesis testing the Tukey post-hoc test was performed. A *p*-value of <0.05 was considered as statistically significant.

## Results

The animals tolerated the irradiation and heparin administration well. No treatment related adverse effects such as weight loss or changes in food uptake or behavior were observed.

### Effect of heparin treatment on the epithelial morphology and proliferation

**Fig. 2:**
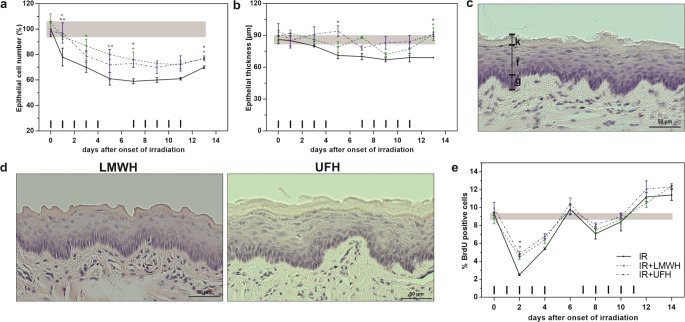
Effect of heparin treatment on epithelial morphology and cell proliferation. Epithelial cell number (**a**), thickness (**b**) and proliferation (e) were assessed over a course of two weeks of fractionated irradiation with 3 Gy (10 × 3 Gy) per fraction, alone or in combination with daily doses of UFH or LMWH (40 and 200 U/mouse/day, respectively) and are represented as the sum of the germinal, functional and keratin layer values (**c**). Pre-treatment with either LMWH or UFH did not alter the epithelial morphology (**d**) compared to the control specimen (**c**). The *shaded area* displays mean ±1 SD of the corresponding control experiment (irradiation alone). The fractionation protocol is indicated on top of the x‑axis. Histophotographs: eosin/hematoxylin staining. *p < 0.05, **p < 0.005; scale bar = 50 µm; *n* = 3. *UFH* unfractionated heparin; *LMWH* low-molecular-weight heparin; *IR* irradiation

During the first experimental week, the fractionated irradiation successively reduced the total number of epithelial cells to a minimum of 60% at day 5 (Fig. 2a). The onset of repopulation in the second irradiation week stabilized the epithelial cell numbers before they began to increase after the last fraction on day 11. Still, the total cell number did not reach the initial value. A similar pattern was observed for the thickness of the epithelium composed of the germinal, functional and keratin layers (Fig. 2b and c) and comprising 86 ± 4 µm in untreated animals. Irradiation alone led to a slight thinning of the epithelium during the first irradiation week. With the onset of repopulation the epithelial thickness stabilized but did not reach the baseline values after the cessation of irradiation. Pre-treatment with heparins starting three days before the first fraction neither provoked elevated epithelial cell numbers nor increased epithelial thickness (Fig. 2d). Systemic treatment with heparins led to significantly higher cell numbers throughout the course of irradiation, e. g. on day 1 (*p* = 0.0068 for UFH and *p* = 0.0207 for LMWH), day 3 (*p* = 0.0143 for UFH), day 5 (*p* = 0.0068 for LMWH), day 7 (*p* = 0.0430 for UFH and *p* = 0.000 for 0143) and day 13 (*p* = 0.0143 for UFH and *p* = 0.0299 for LMWH). The epithelial thickness was mostly unaffected by the heparin application, except for day 5 (*p* = 0.0244 for UFH) and day 13. Here, treatment with both UFH and LMWH provoked significantly elevated epithelial thickness, even restoring the baseline values (*p* = 0.0313).

Fractionated irradiation rapidly annihilated epithelial cell proliferation, represented by a strong decrease of BrdU incorporation in the control specimen from 9.3% on day 0 to 2.5% on day 2 (Fig. 2e). The proliferation was restored to baseline values by day 6. The onset of repopulation in the beginning of the second irradiation week successively further increased the proliferation to 11.2% on day 12. Additional treatment with both UFH and LMWH led to significantly higher fraction of proliferating cells on day 2 (5%; *p* = 0.0209 and *p* = 0.0448, respectively) but had no effect on the epithelial proliferation throughout the rest of the study course.

### Effect of heparin treatment on the expression of cell junction molecules—adherence junctions

**Fig. 3:**
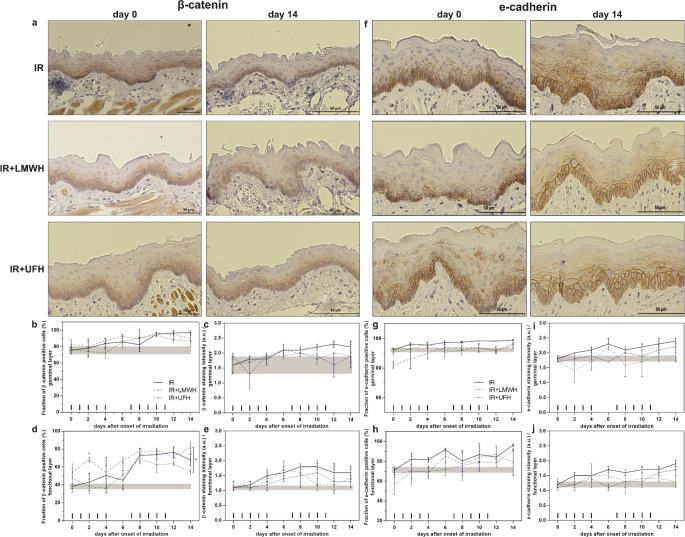
Expression pattern of *β-catenin* (**a**) and *e-cadherin* (**f**) in response to fractionated irradiation without or with additional heparin treatment. Histophotographs illustrate localization and representative marker staining on days 0 and 14 after irradiation alone or in combination with heparin application (**a** and **f**). Fractions of β‑catenin and e‑cadherin positive epithelial cells (**b**,**d** and **g**,**h**) and the respective staining intensity (**c**,**e** and **l**,**j**) were analyzed in the germinal and functional epithelial layers after fractionated irradiation alone or with additional daily UFH or LMWH treatment (40 and 200 U/mouse/day, respectively). The *shaded area* displays mean ±1 SD of the corresponding control experiment (irradiation alone). The fractionation protocol is indicated on top of the x‑axis. *p < 0.05, **p < 0.005; *n* = 3. Scale bar = 50 µm. *UFH* unfractionated heparin; *LMWH* low-molecular-weight heparin; *IR* irradiation

The fraction of β‑catenin positive cells accounted for around 76% in the proliferative germinal layer and 39% in the functional layer; both layers featured an intermediate staining intensity of 1.1–1.6 a.u (Fig. 3b–e). As represented in Fig. 3a, the staining of β‑catenin was restricted to the cell membrane and could be attributed to individual cells. Fractionated irradiation gradually increased the expression of membrane-bound β‑catenin to almost 100% in the germinal layer on day 14; in the functional layer, the fraction of β‑catenin positive cells drastically increased in the beginning of the second irradiation week and almost doubled, with a maximum on day 12. Also the staining intensity gradually increased over the course of two weeks of fractionation in the germinal and functional epithelium compartments, reaching a maximum of 2.3 a.u on day 12 and 1.8 a.u on day 10, respectively. Pre-treatment with heparins over three days before the first fraction slightly elevated the marker positive cells in the functional tissue compartment. Additional systemic treatment with heparins over the two weeks of fractionated irradiation had no significant impact on β‑catenin expression in the germinal layer. When animals were treated with UFH, the expression levels of β‑catenin were slightly higher than in control animals but did not reach statistical significance. The staining intensity was also mostly unaffected. In the functional layer, application of UFH provoked significantly elevated levels of β‑catenin on day 2 (*p* = 0.0446). LMWH treated animals showed a lower fraction of β‑catenin positive cells; on the last day of analysis, day 14, the difference between the heparin medications reached statistical significance favoring UFH application (*p* = 0.0171).

**Fig. 4:**
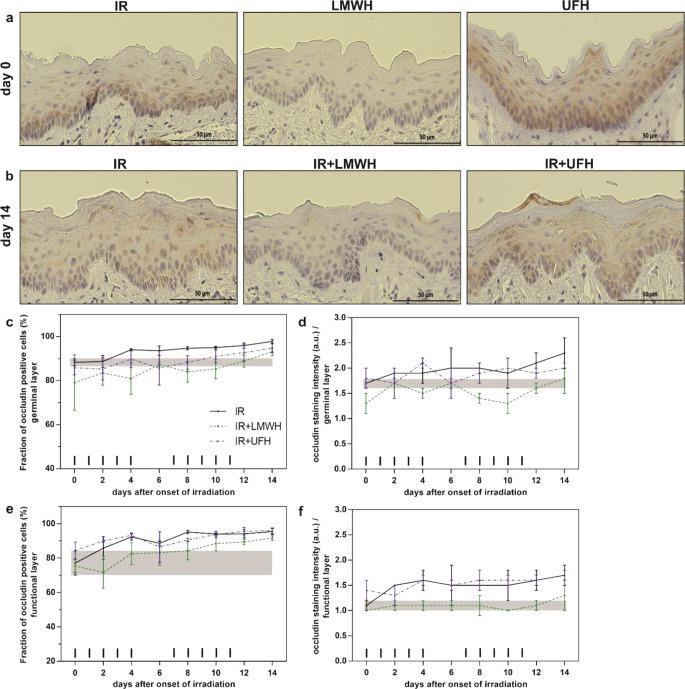
Expression pattern of *occludin* in response to fractionated irradiation without or with additional heparin treatment. Histophotographs illustrate localization and representative marker staining on days 0 (**a**) and 14 (**b**) after irradiation alone or in combination with heparin application. Fraction of occludin positive epithelial cells (**c** and **e**) and the respective staining intensity (**d** and **f**) were analyzed in the germinal and functional epithelial layers after fractionated irradiation alone or with additional daily UFH or LMWH treatment (40 and 200 U/mouse/day, respectively). The *shaded area* displays mean ±1 SD of the corresponding control experiment (irradiation alone). The fractionation protocol is indicated on top of the x‑axis. *p < 0.05, **p < 0.005; *n* = 3. Scale bar = 50 µm. *UFH* unfractionated heparin; *LMWH* low-molecular-weight heparin; *IR* irradiation

In the control specimen, 92% of germinal and 71% of functional cells featured a positive membrane staining for e‑cadherin with an intermediate staining intensity of 1.2–1.8 a.u (Fig. 3f–j). Fractionated irradiation increased the expression of this cell adhesion marker to almost 100% in both tissue compartments. Also the staining intensity increased to a maximum of 2.3 a.u on day 6 in the germinal layer and 1.9 a.u on day 14 in the functional layer. Heparin pre-treated animals showed a slightly decreased fraction of e‑cadherin positive cells in both compartments. Fractionated irradiation gradually increased the expression of e‑cadherin in those animals, but additional treatment with heparins provoked a decreased expression of e‑cadherin compared to the control animals. This was evident by a lower fraction of marker positive cells and decreased staining intensity, in both germinal and functional epithelial layers. On the contrary to the expression levels of β‑catenin, both heparin medications had a comparable impact on the e‑cadherin expression pattern.

### Effect of heparin treatment on the expression of cell junction molecules—tight junctions

In untreated animals, the fraction of occludin-positive cells accounted for 88% in the germinal and 77% in functional cell layer; the corresponding staining intensity was 1.7 a.u and 1.1 a.u (Fig. 4c–e). Fractionated irradiation elevated the marker expression until day 5 and, during the second irradiation week, the values almost reached 100% on day 14. The corresponding staining intensity increased gradually in response to irradiation to a maximum of 2.3 a.u in germinal and 1.7 a.u. in the functional tissue compartments. Pre-treatment with heparins provoked slightly reduced expression of occludin in both layers. Additional treatment with UFH had no impact neither on the fraction of occludin positive cells nor on the respective staining intensity. When fractionated irradiation was combined with LMWH treatment, the expression of occludin was reduced, evident by a lower fraction of marker positive cells and decreased staining intensity in both germinal and functional epithelial layers.

## Discussion

Radiotherapy is inevitably associated with a certain but accepted risk of normal tissue side effects [5]. In the head-and-neck region, conventional radiotherapy provokes development of oral mucositis, a common severe and often dose-limiting condition. It represents a significant issue in oncology, since no effective target-based intervention has been introduced into clinical routine so far [4].

Recently, we described a muco-protective effect of systemic heparin treatment on radiation-induced oral mucositis in a pre-clinical model [12]. In this study, we tested varying application intervals for UFH and LMWH in combination with single dose or fractionated irradiation regimes. Heparin treatment resulted in lower incidence of radiation-induced mucositis, prolonged the latent time until the ulcer development and reduced the mucositis duration. These effects were particularly pronounced when fractionated irradiation over two weeks was combined with daily heparin application starting three days before the first fraction until the day of the last fraction, i. e. day 11. Consequently, this therapy combination was chosen for the present study, focusing on the underlying mechanisms of the observed radio-protective effect of heparin on normal oral epithelium.

Fractionated irradiation over two weeks had a major effect on the epithelial morphology by rapidly downregulating the proliferative capacity in the germinal epithelial layer resulting in reduced total epithelial cell number and thickness. With the onset of the regenerative tissue response to radiation in the beginning of the second week of fractionation, called repopulation, epithelial cell number and thickness stabilized for the rest of study period and cell proliferation recovered, even beyond background values. These morphological changes were accompanied by elevated expression of cell junction proteins, most likely in order to counteract the continuous shedding of superficial cells and help maintaining the mucosal integrity.

When fractionated irradiation was accompanied by daily treatment with heparins, the radiation response of normal mucosa was modified. Over the course of two weeks, we observed significantly higher epithelial cell numbers and slightly increased thickness of the epithelium. The loss of epithelial cells in response to radiation was clearly less prominent and the cell numbers stabilized earlier, compared to untreated animals. These findings might explain the observation of the prolonged latent time and reduced ulcer duration in our previous study. The epithelial proliferation was mostly unaffected by systemic heparin treatment, except for day 2. Here, markedly more cells retained their proliferating capacity. It seems that heparin application provokes an earlier onset of the adaptive repopulation leading to increased radiation tolerance with the increasing overall treatment time. This might explain why the strongest muco-protective potential in our previous study was observed when irradiation was applied over two weeks. Potentially, the earlier onset of the regenerative response provoked by application of heparin leads to a decreased incidence of ulcerations. As described by Dörr et al., three major mechanisms are responsible for repopulation: acceleration of stem cell proliferation, asymmetry loss of stem cell divisions and abortive divisions of sterilized cells [16]. The pronounced muco-mitigative effect of heparin application might be related to interactions with all of these complex mechanisms.

Concerning the expression of cell junction proteins, only the levels of β‑catenin could be elevated by additional heparin treatment; the expression of e‑cadherin and occludin were marginally changed. It seems obvious that the interplay of heparin with the analyzed cell junction molecules plays rather a minor role in the muco-protective effect. The reason for maintenance of the mucosal integrity relies mostly on the higher fraction of surviving cells that keep their proliferative capacity. Important to mention is also the fact that the potential muco-protective effect of heparin is not based on stimulation of epithelial proliferation.

Both heparin preparations used in this study are approved for clinical applications and are widely used in clinical practice mostly because of their anticoagulant properties. A large body of literature exists stating that heparins, besides anti-coagulation, might play an important role in biological processes such as proliferation, infection, immune response, cell adhesion and inflammation [15]. Since the manifestation of radiation-induced oral mucositis is a dynamic event involving multiple molecular and cellular processes in all compartments of the mucosa, further work is required in order to characterize the underlying mechanisms of heparin’s muco-protective potential in detail. Additionally, studies suggest that glycans play a crucial role in all physiological steps of tumor progression by regulating tumor proliferation, invasion, metastasis and angiogenesis [17]. In vivo studies could show that heparins, especially LMWH, possess anti-tumor and anti-metastatic potential and prolonged survival of cancer patients [18]. To conclude, it seems that heparin treatment supportive to anti-cancer radiotherapy has a positive protective effect on the normal oral epithelium and potentially anti-tumor effects. These properties render heparins perfect candidates for further clinical studies.

## Conclusion

The present study demonstrates that the muco-protective potential of systemic heparin treatment on radiation-induced oral mucositis is partially based on the interaction with the repopulation processes, resulting in an earlier onset of this adaptive response. Additionally, we could highlight that the concomitant heparin treatment did not stimulate the proliferation of the normal tissue. Since both heparin preparations are already approved for clinical use, they seem to be promising drugs for future clinical studies. Nevertheless, further studies are necessary to identify and characterize additional biological mechanisms underlying the modulatory effects and questioning the proliferation stimulating effect on the malignant cells by heparins (manuscripts in preparation).
