# Publisher Correction to: Heparin treatment mitigates radiation-induced oral mucositis in mice by interplaying with repopulation processes

**DOI:** 10.1007/s00066-019-01560-4

**Published:** 2019-12-23

**Authors:** Springer Berlin Heidelberg

**Affiliations:** http://www.springer.com/66

**Correction to:**

**Strahlenther Onkol 2018**

10.1007/s00066-018-01423-4

The article was initially published with incorrect copyright information or changed later to incorrect copyright information.

Upon publication of this Correction, the copyright of the article changed to “The Author(s)”.

The original article has been corrected.

